# Supplementary material for: Identification of the BcLEA Gene Family and Functional Analysis of the BcLEA73 Gene in Wucai (Brassica campestris L.)
Source: Genes (Basel). 2023 Feb 5;14(2):415. doi: 10.3390/genes14020415 (PMC9957401; doi:10.3390/genes14020415)
Supplement: Supplementary file 1 [file genes-14-00415-s001.zip › genes-2150565-supplementary.pdf]

Table S1. Identification and analysis of the physicochemical properties of the *BrLEA* gene family members.

Gene Name	Gene ID	Chr	Family	aa Len	GRAVY	Mw/Da	pI	Subcelluar location	Intron number
BrLEA1	*Bra011874.1*	A01	Dehydrin	149	-0.863	15928.4	5.65	Cytoplasm	1
BrLEA2	*Bra033520.1*	A01	LEA_3	99	-0.4	10582.01	9.72	Nucleus	1
BrLEA3	*Bra031399.1*	A01	LEA_2	150	-0.663	17469.06	9.61	Chloroplast	0
BrLEA4	*Bra021311.1*	A01	LEA_2	223	-0.399	25914	10.04	Chloroplast	0
BrLEA5	*Bra038671.1*	A01	LEA_2	211	0.108	23726.39	9.68	Cell membrane	0
BrLEA6	*Bra038669.1*	A01	LEA_2	234	0.033	26508.87	8.99	Nucleus	0
BrLEA7	*Bra028562.1*	A02	LEA_2	209	0.113	23613.3	9.26	Cell membrane	0
BrLEA8	*Bra020222.1*	A02	LEA_2	132	-0.434	15043.08	9.3	Chloroplast	0
BrLEA9	*Bra022642.1*	A02	SMP	173	-0.357	18379.46	5.88	Nucleus	1
BrLEA10	*Bra039737.1*	A02	LEA_2	248	-0.212	27627.95	9.47	Nucleus	1
BrLEA11	*Bra008242.1*	A02	Dehydrin	194	-1.352	21840.38	5.52	Nucleus	1
BrLEA12	*Bra020604.1*	A02	SMP	181	-0.481	18366.26	4.76	Nucleus	2
BrLEA13	*Bra031809.1*	A02	Dehydrin	192	-1.021	18844.17	7.14	Cytoplasm	2
BrLEA14	*Bra005891.1*	A03	LEA_2	235	-0.105	26245.37	9.41	Chloroplast	1
BrLEA15	*Bra005911.1*	A03	LEA_1	159	-0.818	16180.83	8.93	Nucleus	1
BrLEA16	*Bra029085.1*	A03	SMP	177	-0.351	18691.81	5.43	Nucleus	1
BrLEA17	*Bra022950.1*	A03	LEA_6	71	-1.238	7581.23	6.03	Nucleus	0
BrLEA18	*Bra000173.1*	A03	LEA_5	88	-1.659	9630.38	5.88	Nucleus	1
BrLEA19	*Bra000339.1*	A03	LEA_2	777	-0.135	84314.53	9.02	Chloroplast	10
BrLEA20	*Bra000414.1*	A03	LEA_2	166	0.03	17823.38	4.81	Chloroplast	1
BrLEA21	*Bra000519.1*	A03	LEA_2	228	-0.35	25831.24	9.45	Cell membrane	0
BrLEA22	*Bra000526.1*	A03	LEA_2	256	-0.05	28462.98	9.41	Chloroplast	0
BrLEA23	*Bra000884.1*	A03	LEA_3	94	-0.349	10095.53	9.85	Chloroplast	1
BrLEA24	*Bra001869.1*	A03	SMP	239	-0.38	25385.45	5.7	Nucleus	1
BrLEA25	*Bra012801.1*	A03	LEA_2	208	0.152	23027.83	9.86	Cell membrane	0
BrLEA26	*Bra014864.1*	A04	LEA_3	122	-0.598	14152.16	9.2	Chloroplast	1
BrLEA27	*Bra033428.1*	A04	LEA_2	208	0.144	23136.02	9.89	Chloroplast	0
BrLEA28	*Bra028103.1*	A04	LEA_2	255	-0.2	28751.42	9.86	Chloroplast	1
BrLEA29	*Bra034321.1*	A04	LEA_2	260	-0.179	28595.09	9.86	Chloroplast	0
BrLEA30	*Bra034341.1*	A04	LEA_2	238	-0.262	26639.28	9.49	Cell membrane	0
BrLEA31	*Bra021869.1*	A04	LEA_6	72	-1.174	7627.3	5.21	Nucleus	0
BrLEA32	*Bra017276.1*	A04	LEA_2	208	0.064	23279.91	9.51	Chloroplast	0
BrLEA33	*Bra017272.1*	A04	LEA_2	227	-0.062	25700.98	9.88	Cell membrane	0

BrLEA34	*Bra017229.1*	A04	LEA_4	398	-1.065	43386.25	6.02	Cell wall	2
BrLEA35	*Bra016868.1*	A04	LEA_4	679	-1.088	72972.61	5.98	Cell wall	1
BrLEA36	*Bra037669.1*	A04	LEA_2	506	-0.399	55465.06	8.85	Chloroplast	3
BrLEA37	*Bra039288.1*	A04	LEA_2	166	0.013	18119.88	4.5	Chloroplast	1
BrLEA38	*Bra004564.1*	A05	LEA_2	220	0.047	24269.55	9.98	Chloroplast	0
BrLEA39	*Bra004565.1*	A05	LEA_2	166	0.093	17725.34	4.81	Chloroplast	1
BrLEA40	*Bra004981.1*	A05	LEA_5	84	-1.704	9264.98	6.74	Nucleus	1
BrLEA41	*Bra005256.1*	A05	LEA_4	399	-0.97	43565.34	5.74	Cell wall	3
BrLEA42	*Bra005340.1*	A05	LEA_2	233	-0.082	26086.31	9.45	Chloroplast	0
BrLEA43	*Bra005353.1*	A05	LEA_1	98	-1.115	10666.89	9.22	Nucleus	0
BrLEA44	*Bra018556.1*	A05	LEA_3	93	-0.482	10112.56	9.99	Chloroplast	1
BrLEA45	*Bra039956.1*	A05	SMP	262	-0.239	26692.5	4.71	Nucleus	2
BrLEA46	*Bra034822.1*	A05	LEA_2	212	0.021	23866.49	9.65	Chloroplast	0
BrLEA47	*Bra018649.1*	A06	LEA_2	207	-0.153	23555.65	10.29	Chloroplast	0
BrLEA48	*Bra019628.1*	A06	LEA_4	451	-1.141	48554.47	5.2	Cell wall	2
BrLEA49	*Bra025819.1*	A06	Dehydrin	271	-1.508	31018.16	5	Nucleus	1
BrLEA50	*Bra019437.1*	A06	LEA_2	183	0.148	20019.22	6.65	Nucleus	0
BrLEA51	*Bra009983.1*	A06	SMP	192	-0.515	19473.41	4.76	Nucleus	2
BrLEA52	*Bra025130.1*	A06	LEA_4	201	-0.429	22054.03	5.4	Nucleus	1
BrLEA53	*Bra024907.1*	A06	LEA_2	228	0.116	24568.58	9.18	Chloroplast	1
BrLEA54	*Bra039616.1*	A07	LEA_4	480	-0.921	52652.67	6.47	Nucleus	1
BrLEA55	*Bra012230.1*	A07	Dehydrin	220	-1.399	24837.39	5.11	Nucleus	1
BrLEA56	*Bra003175.1*	A07	LEA_2	200	0.151	32517.04	10.24	Nucleus	2
BrLEA57	*Bra003732.1*	A07	Dehydrin	194	-1.308	21755.35	5.47	Nucleus	1
BrLEA58	*Bra015779.1*	A07	Dehydrin	195	-1.455	22044.52	5.45	Nucleus	1
BrLEA59	*Bra014346.1*	A08	LEA_2	264	-0.057	28400.74	9.45	Cell membrane	1
BrLEA60	*Bra013992.1*	A08	Dehydrin	257	-1.645	28978	6.17	Nucleus	1
BrLEA61	*Bra038061.1*	A08	LEA_3	57	-0.912	6614.4	4.94	Nucleus	0
BrLEA62	*Bra035491.1*	A08	LEA_1	132	-1.109	14933.95	9.68	Nucleus	1
BrLEA63	*Bra010561.1*	A08	LEA_4	487	-0.923	52431.94	5.63	Cell wall	2
BrLEA64	*Bra030494.1*	A08	LEA_2	151	0.075	16404.92	4.72	Chloroplast	1
BrLEA65	*Bra036272.1*	A09	LEA_3	94	-0.299	10088.55	9.99	Chloroplast	1
BrLEA66	*Bra036112.1*	A09	SMP	191	-0.414	19600.77	4.99	Nucleus	2
BrLEA67	*Bra037177.1*	A09	Dehydrin	144	-1.36	15139.46	8.81	Cytoplasm	2
BrLEA68	*Bra027969.1*	A09	LEA_2	269	-0.217	30181.66	10.24	Chloroplast	0
BrLEA69	*Bra029567.1*	A09	LEA_2	231	-0.158	26511.59	10.45	Mitochondrio n	0
BrLEA70	*Bra023278.1*	A09	LEA_1	133	-0.878	14507.53	9.24	Nucleus	1
BrLEA71	*Bra036843.1*	A09	Dehydrin	134	-0.887	13834.22	9.19	Cytoplasm	1
BrLEA72	*Bra006927.1*	A09	LEA_2	208	0.139	23087.91	9.93	Cell wall	0
BrLEA73	*Bra039946.1*	A09	LEA_6	82	-1.055	8433.11	4.72	Nucleus	0
BrLEA74	*Bra031192.1*	A09	Dehydrin	183	-1.016	19183.88	6.49	Cytoplasm	1
BrLEA75	*Bra031615.1*	A09	LEA_2	207	-0.103	23424.42	10.13	Chloroplast	1

BrLEA76	*Bra033377.1*	A10	SMP	283	-0.543	32586.25	8.89	Chloroplast	7
BrLEA77	*Bra033375.1*	A10	SMP	177	-0.54	18185.98	4.53	Nucleus	1
BrLEA78	*Bra033350.1*	A10	LEA_3	98	-0.229	10298.59	8.03	Chloroplast	1
BrLEA79	*Bra009184.1*	A10	LEA_2	237	-0.17	26493.54	9.27	Chloroplast	0
BrLEA80	*Bra009225.1*	A10	LEA_1	159	-0.789	16286.99	9.22	Nucleus	1
BrLEA81	*Bra035001.1*	Scaffold000100	LEA_2	206	0.056	23148.61	7.09	Chloroplast	1
BrLEA82	*Bra040894.1*	Scaffold000300	LEA_5	172	-1.635	18997.7	6.17	Nucleus	1
